# Supplementary material for: The Effects of the Steroids 5-Androstenediol and Dehydroepiandrosterone and Their Synthetic Derivatives on the Viability of K562, HeLa, and Wi-38 Cells and the Luminol-Stimulated Chemiluminescence of Peripheral Blood Mononuclear Cells from Healthy Volunteers
Source: Biomolecules. 2024 Mar 19;14(3):373. doi: 10.3390/biom14030373 (PMC10967766; doi:10.3390/biom14030373)
Supplement: Supplementary file 1 [file biomolecules-14-00373-s001.zip › biomolecules-2922073-supplementary.pdf]

Table S1. Crystal data and details of X-ray analysis.

	**2**	**3d**
Formula	$C_{20}H_{34}O_3$	$C_{20}H_{30}O_4S_1$
*F*w	322.47	366.50
colour, habit	colourless, needle	colourless, prism
cryst size (mm)	0.20×0.03×0.02	0.25×0.25×0.20
temperature (K)	100	200
crystal system	orthorhombic	orthorhombic
space group	$P2_12_12_1$	$P2_12_12_1$
a (Å)	9.1865(5)	10.9534(3)
b (Å)	16.3097(8)	11.2324(3)
c (Å)	23.7715(12)	15.4057(4)
β (deg)	90	90
V (Å^3)	3561.7(3)	1895.41(9)
Z	8	4
D_c (g·cm^{-3})	1.203	1.284
μ (mm^{-1})	0.078	0.192
F(000)	1424	792
θ range (deg)	2.12 to 26.98	2.24 to 28.00
refln collcd	58491	27405
indep reflns / R_{int}	7771 / 0.0536	4563 / 0.0299
reflns $I>2\sigma(I)$	7080	4206
No of param	439	346
GooF on F^2	1.111	1.039
R_1 ($I>2\sigma(I)$)	0.0550	0.0348
wR_2(all data)	0.1316	0.0865
Flack parameter	—	0.00(2)
largest diff peak / hole (e·Å^{-3})	0.38 / -0.29	0.22 / -0.27

^{1}H and ^{13}C spectra for the synthesized compounds

2 1H

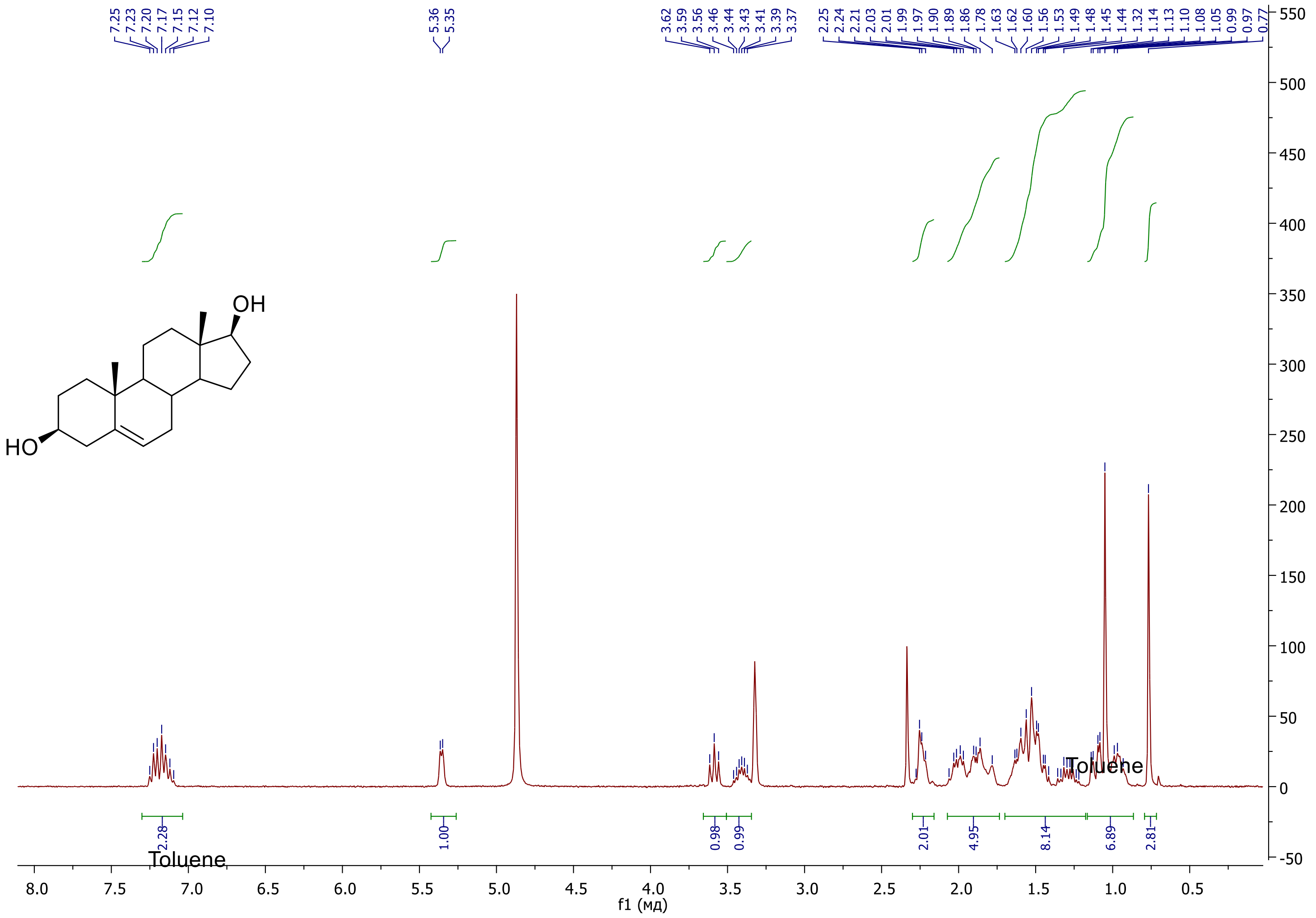

2 13C

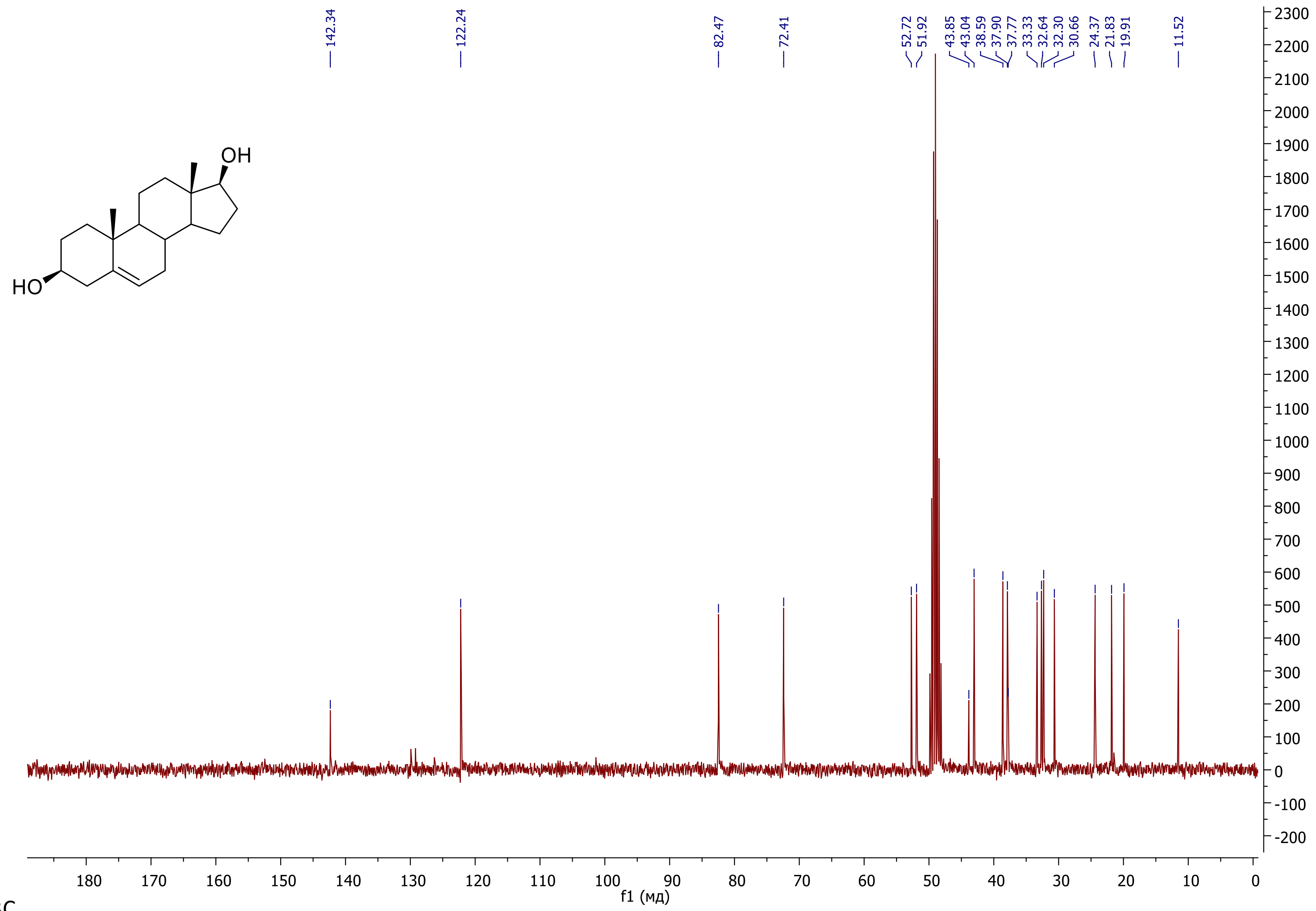

OH
HO
142.34
122.24
82.47
72.41
52.72
51.92
43.85
43.04
38.59
37.90
37.77
33.33
32.64
32.30
30.66
24.37
21.83
19.91
11.52
f1 (мд)

3C

3a 1H

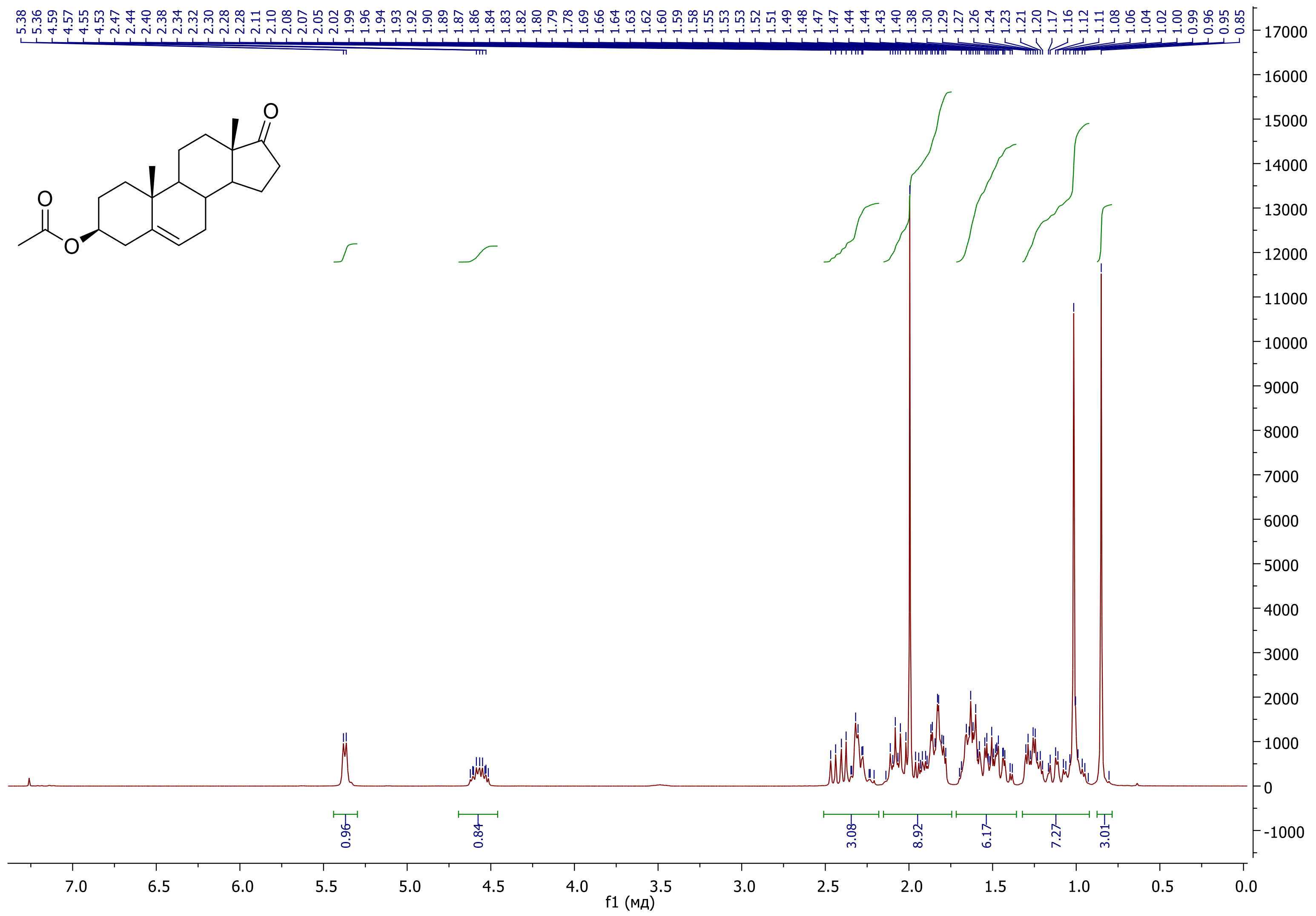

5.38
5.36
4.59
4.57
4.55
4.53
2.47
2.44
2.40
2.38
2.34
2.32
2.30
2.28
2.28
2.11
2.10
2.08
2.07
2.05
2.02
1.99
1.96
1.94
1.93
1.92
1.90
1.89
1.87
1.86
1.84
1.83
1.82
1.80
1.79
1.78
1.69
1.66
1.64
1.63
1.62
1.60
1.59
1.58
1.55
1.53
1.53
1.52
1.51
1.49
1.48
1.47
1.47
1.44
1.44
1.43
1.40
1.38
1.30
1.29
1.27
1.26
1.24
1.23
1.21
1.20
1.17
1.16
1.12
1.11
1.08
1.06
1.04
1.02
1.00
0.99
0.96
0.95
0.85
0.96
0.84
3.08
8.92
6.17
7.27
3.01
f1 (мд)

3a 13C

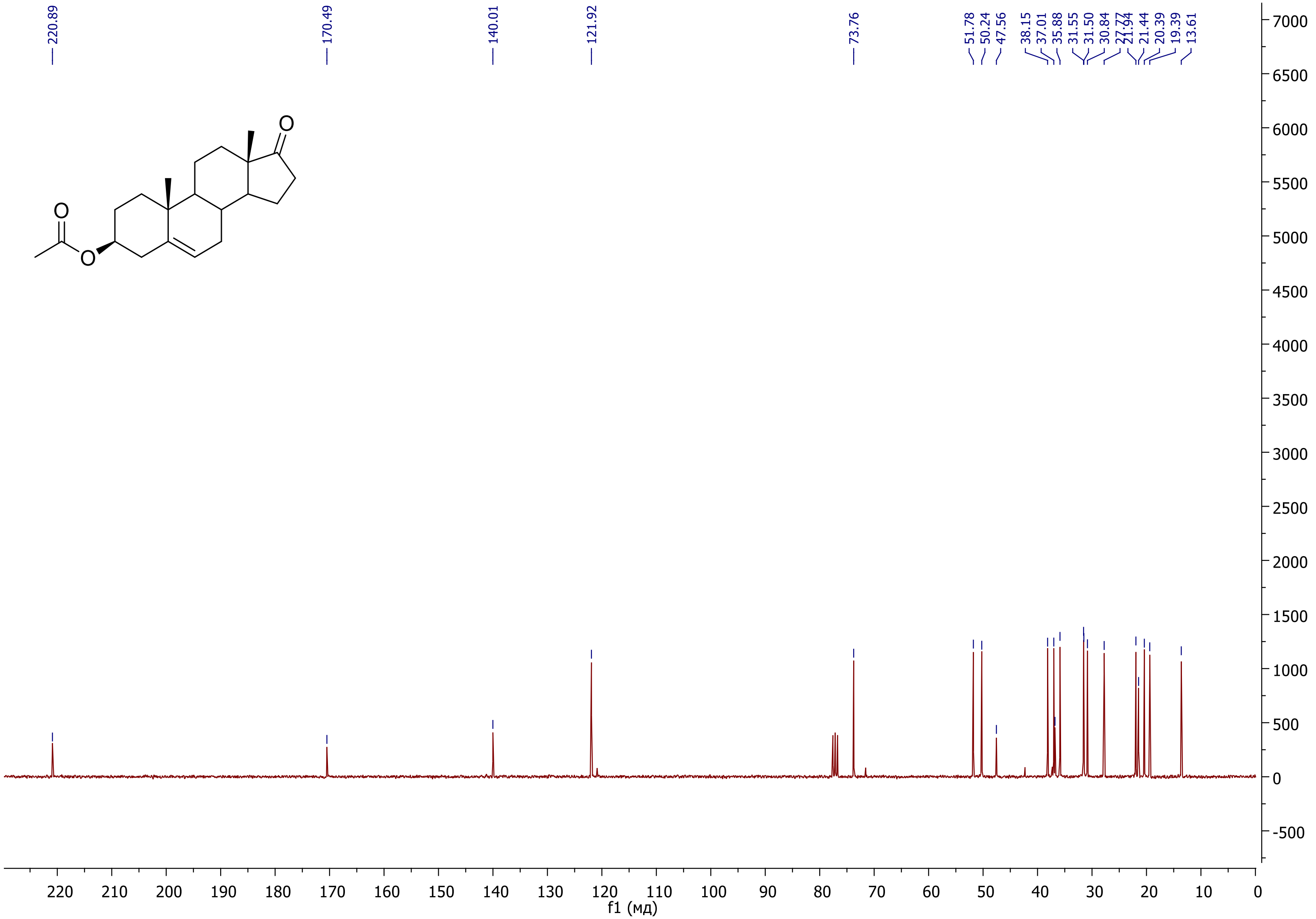

220.89
170.49
140.01
121.92
73.76
51.78
50.24
47.56
38.15
37.01
35.88
31.55
31.50
30.84
27.77
21.94
21.44
20.39
19.39
13.61
O
O
O
7000
6500
6000
5500
5000
4500
4000
3500
3000
2500
2000
1500
1000
500
0
-500
220 210 200 190 180 170 160 150 140 130 120 110 100 90 80 70 60 50 40 30 20 10 0
f1 (мд)

3b 1H

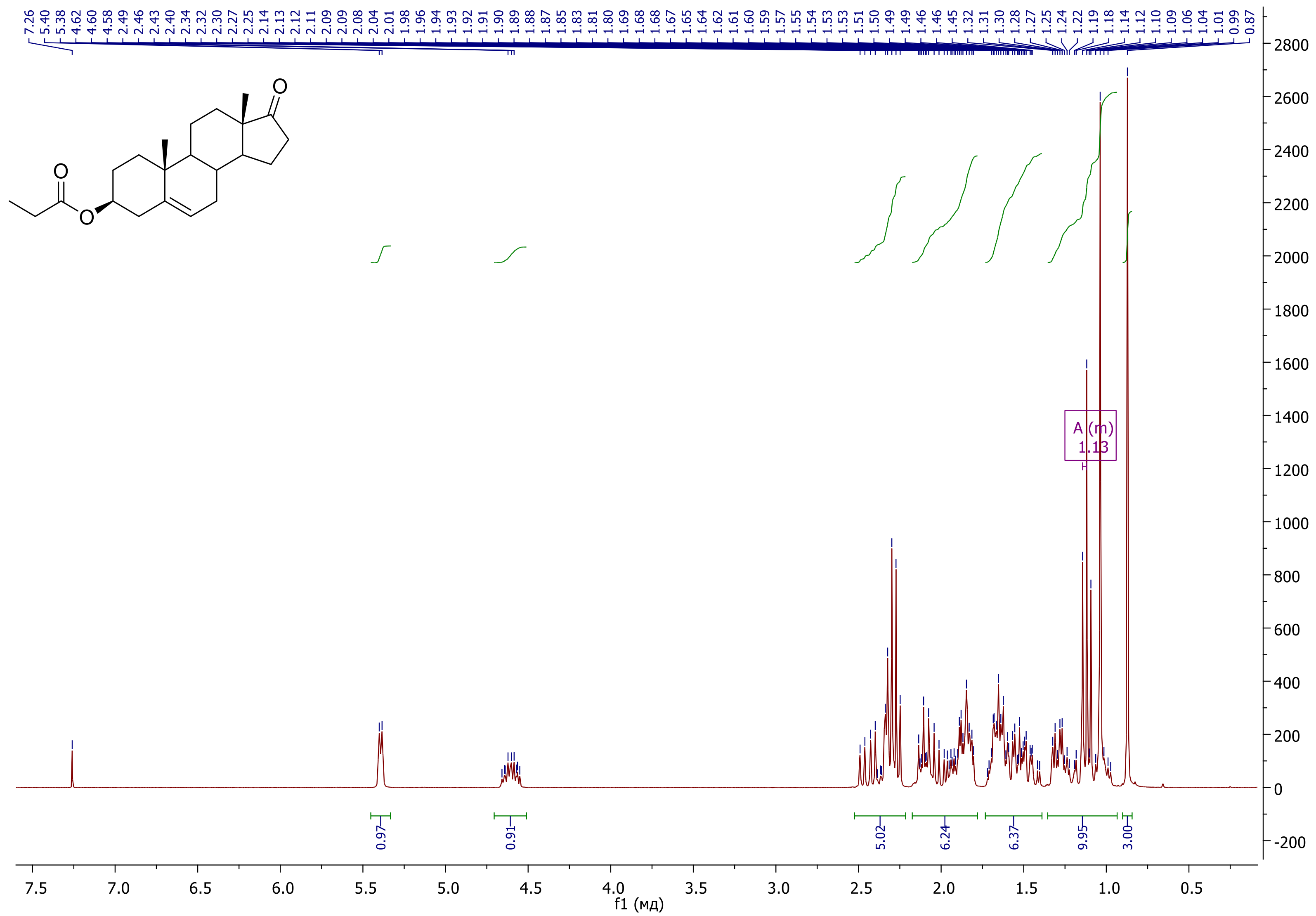

7.26
5.40
5.38
4.62
4.60
4.58
2.49
2.46
2.43
2.40
2.34
2.32
2.30
2.27
2.25
2.14
2.13
2.12
2.11
2.09
2.09
2.08
2.04
2.01
1.98
1.96
1.94
1.93
1.92
1.91
1.90
1.89
1.88
1.87
1.85
1.83
1.81
1.80
1.69
1.68
1.68
1.67
1.65
1.64
1.62
1.61
1.60
1.59
1.57
1.55
1.54
1.53
1.53
1.51
1.50
1.49
1.49
1.46
1.46
1.45
1.32
1.31
1.30
1.28
1.27
1.25
1.24
1.22
1.19
1.18
1.14
1.12
1.10
1.09
1.06
1.04
1.01
0.99
0.87
A (m)
1.13
0.97
0.91
5.02
6.24
6.37
9.95
3.00
f1 (мд)

3b 13C

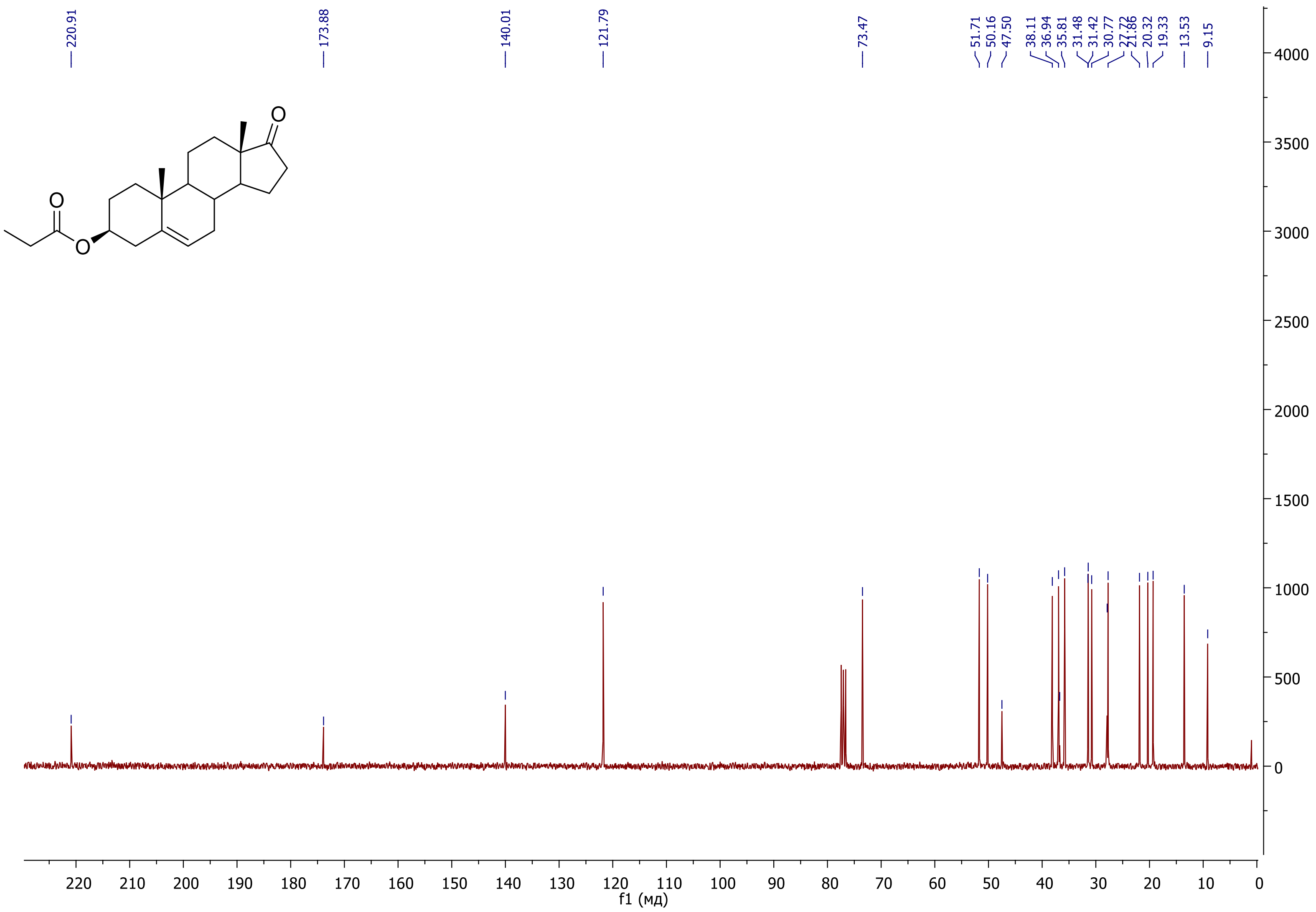

220.91
173.88
140.01
121.79
73.47
51.71
50.16
47.50
38.11
36.94
35.81
31.48
31.42
30.77
27.72
21.86
20.32
19.33
13.53
9.15
4000
3500
3000
2500
2000
1500
1000
500
0
220 210 200 190 180 170 160 150 140 130 120 110 100 90 80 70 60 50 40 30 20 10 0
f1 (мд)

3c 1H

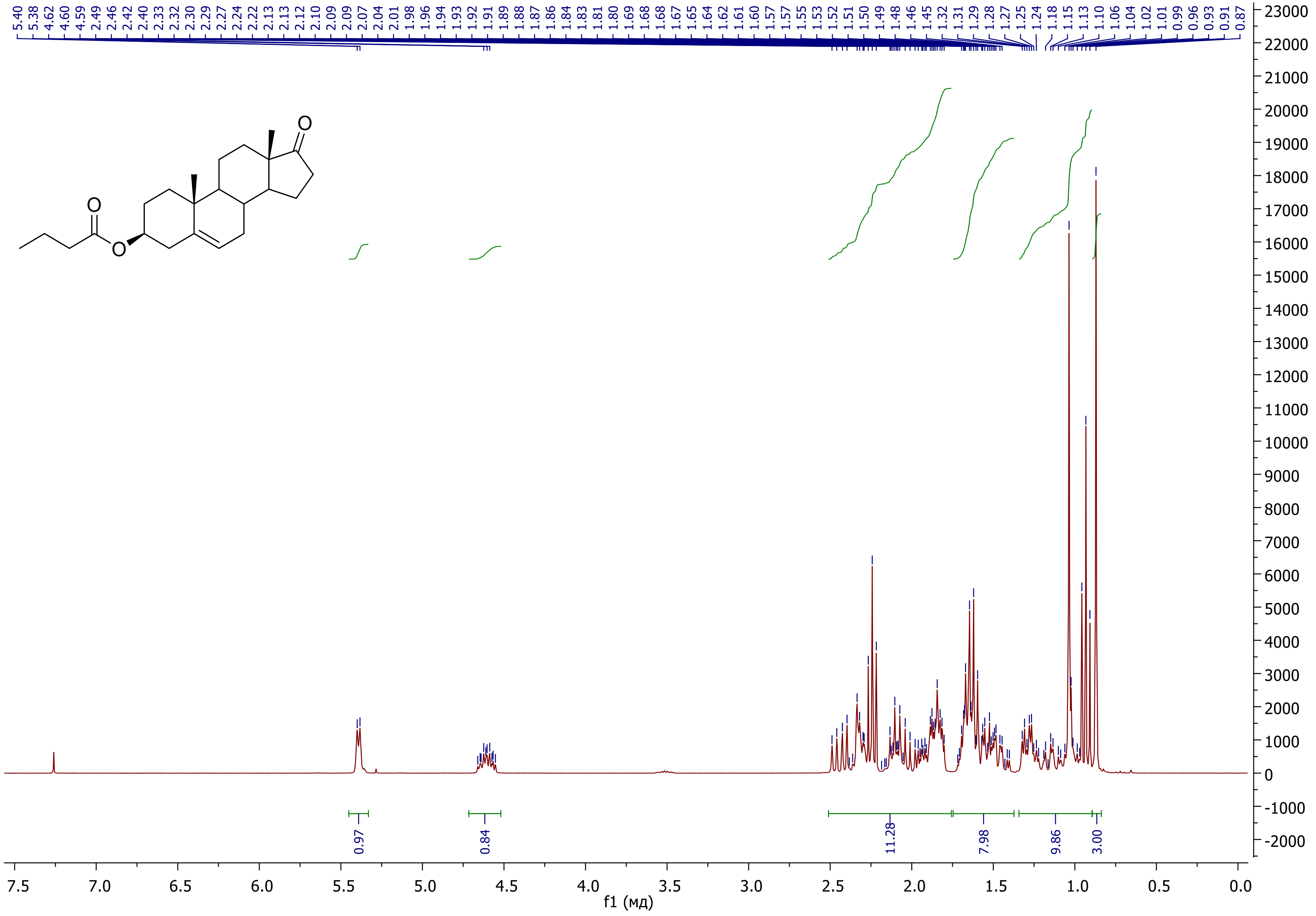

3c 13C

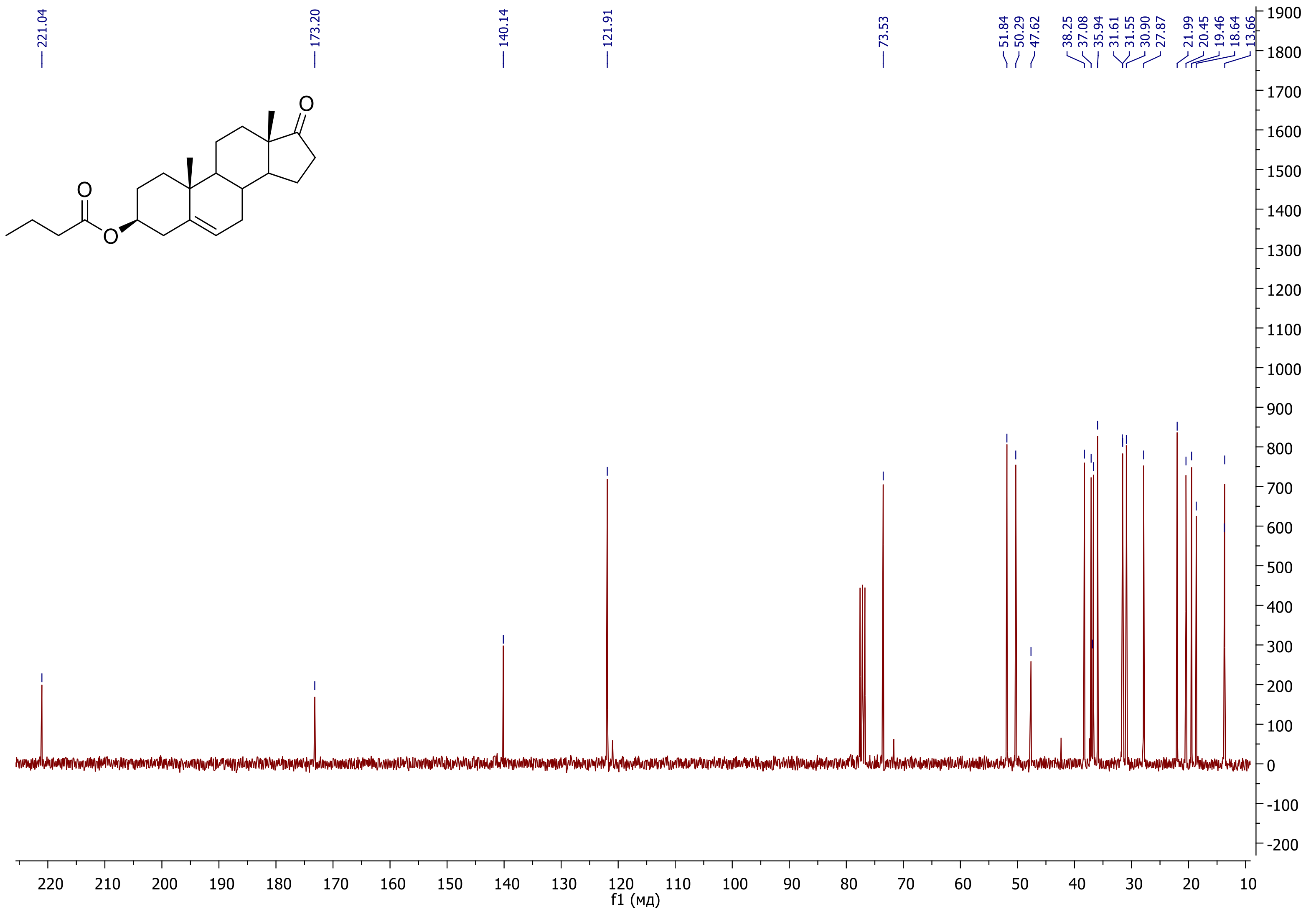

221.04
173.20
140.14
121.91
73.53
51.84
50.29
47.62
38.25
37.08
35.94
31.61
31.55
30.90
27.87
21.99
20.45
19.46
18.64
13.66
f1 (мд)

3d 1H

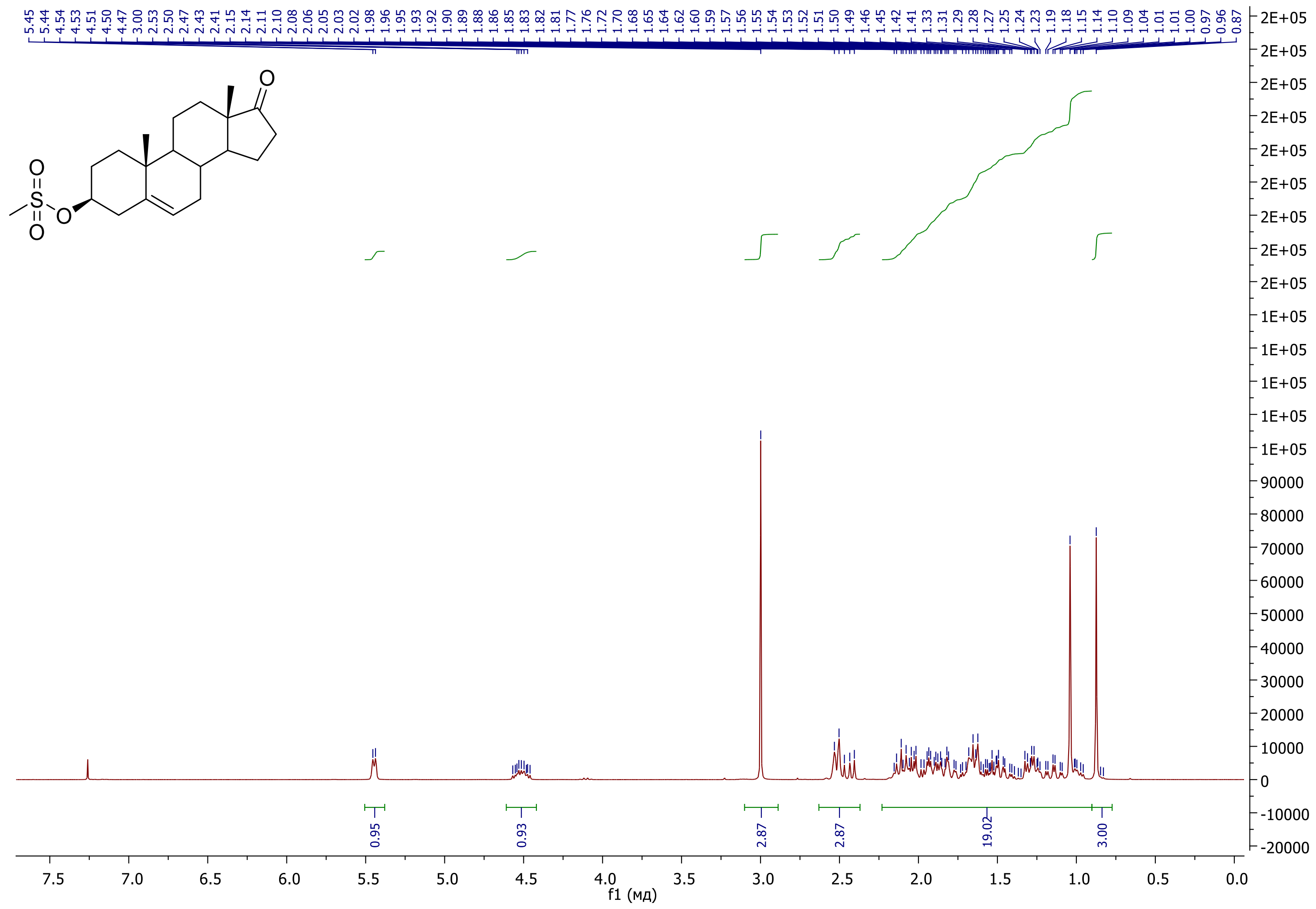

3d 13C

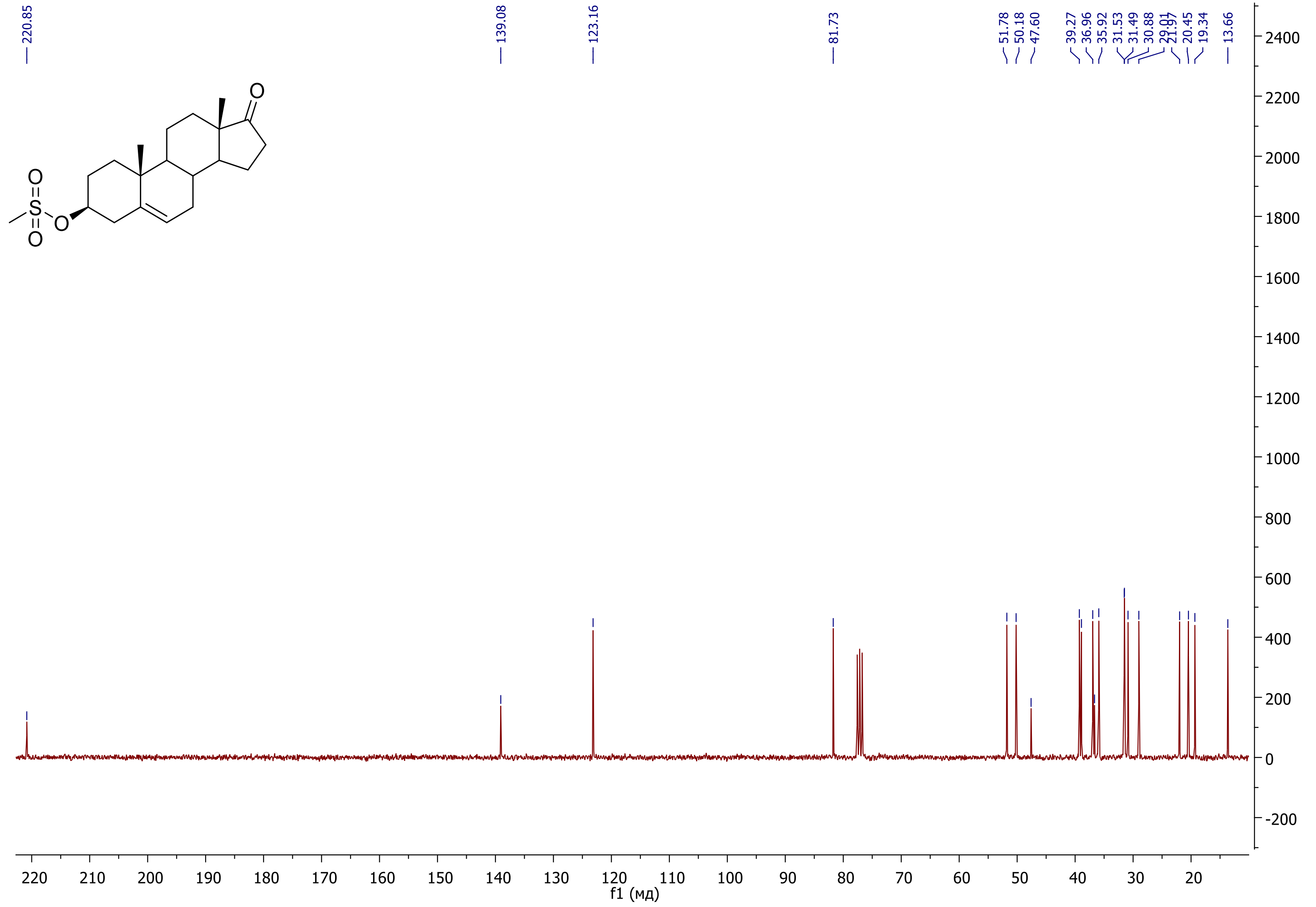
220.85
139.08
123.16
81.73
51.78
50.18
47.60
39.27
36.96
35.92
31.53
31.49
30.88
29.01
21.97
20.45
19.34
13.66
f1 (мд)

4b 1H

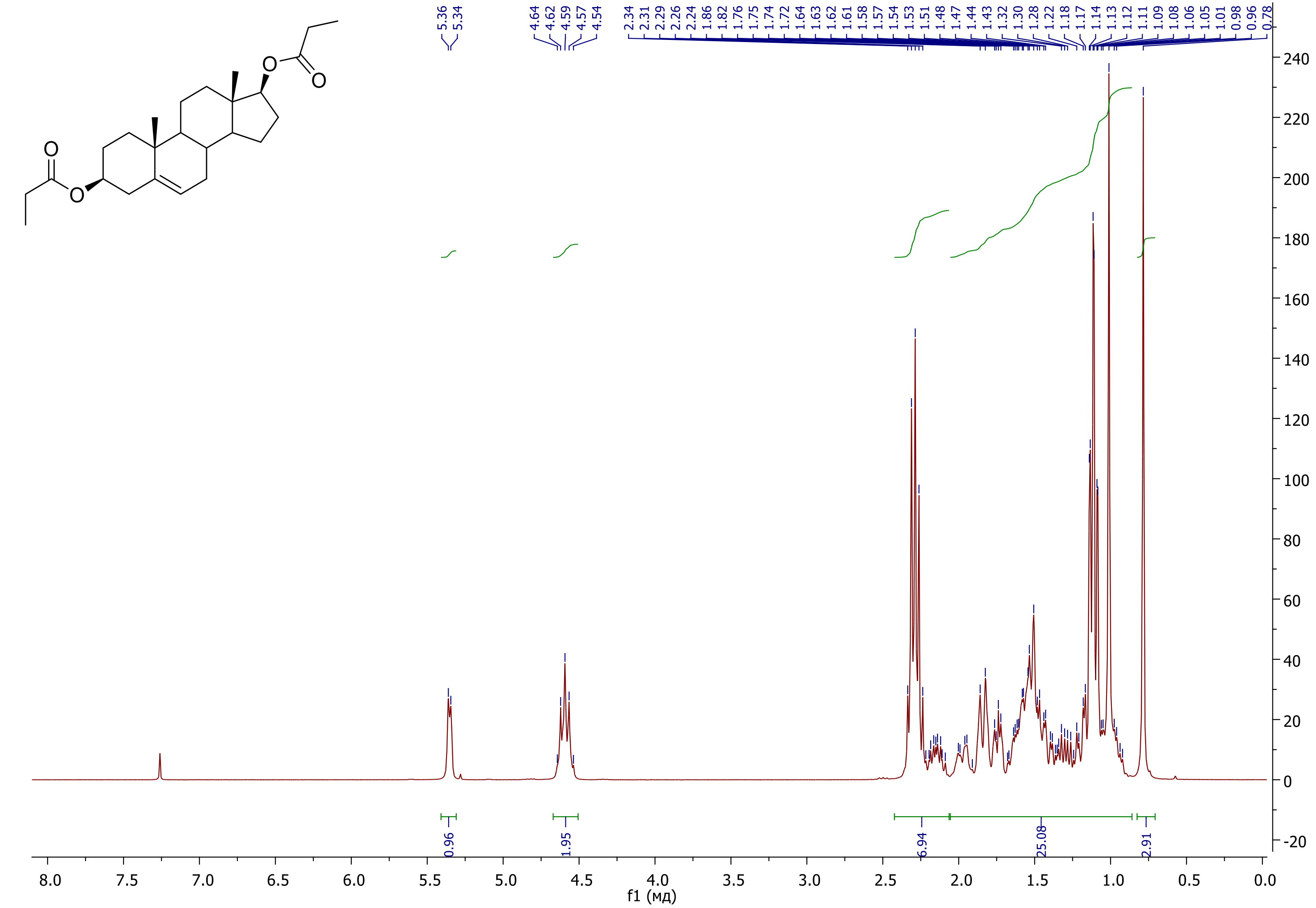

O
O
O
O
5.36
5.34
4.64
4.62
4.59
4.57
4.54
2.34
2.31
2.29
2.26
2.24
1.86
1.82
1.76
1.75
1.74
1.72
1.64
1.63
1.62
1.61
1.58
1.57
1.54
1.53
1.51
1.48
1.47
1.44
1.43
1.32
1.30
1.28
1.22
1.18
1.17
1.14
1.13
1.12
1.11
1.09
1.08
1.06
1.05
1.01
0.98
0.96
0.78
0.96
1.95
6.94
25.08
2.91
8.0 7.5 7.0 6.5 6.0 5.5 5.0 4.5 4.0 3.5 3.0 2.5 2.0 1.5 1.0 0.5 0.0
f1 (мд)
240 220 200 180 160 140 120 100 80 60 40 20 0 -20

4b 13C

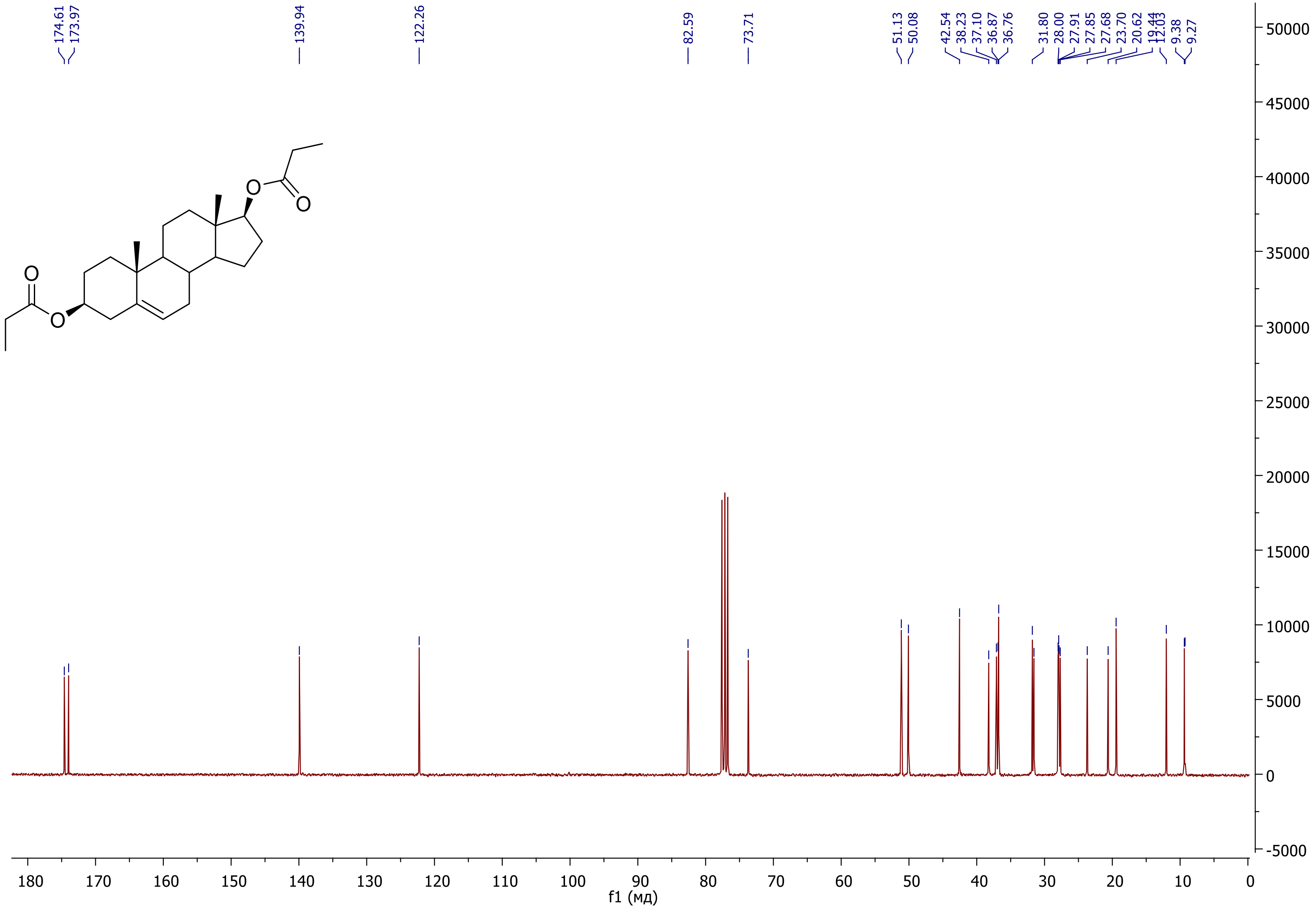

174.61
173.97
139.94
122.26
82.59
73.71
51.13
50.08
42.54
38.23
37.10
36.87
36.76
31.80
28.00
27.91
27.85
27.68
23.70
20.62
19.44
12.03
9.38
9.27
f1 (мд)

4c 1H

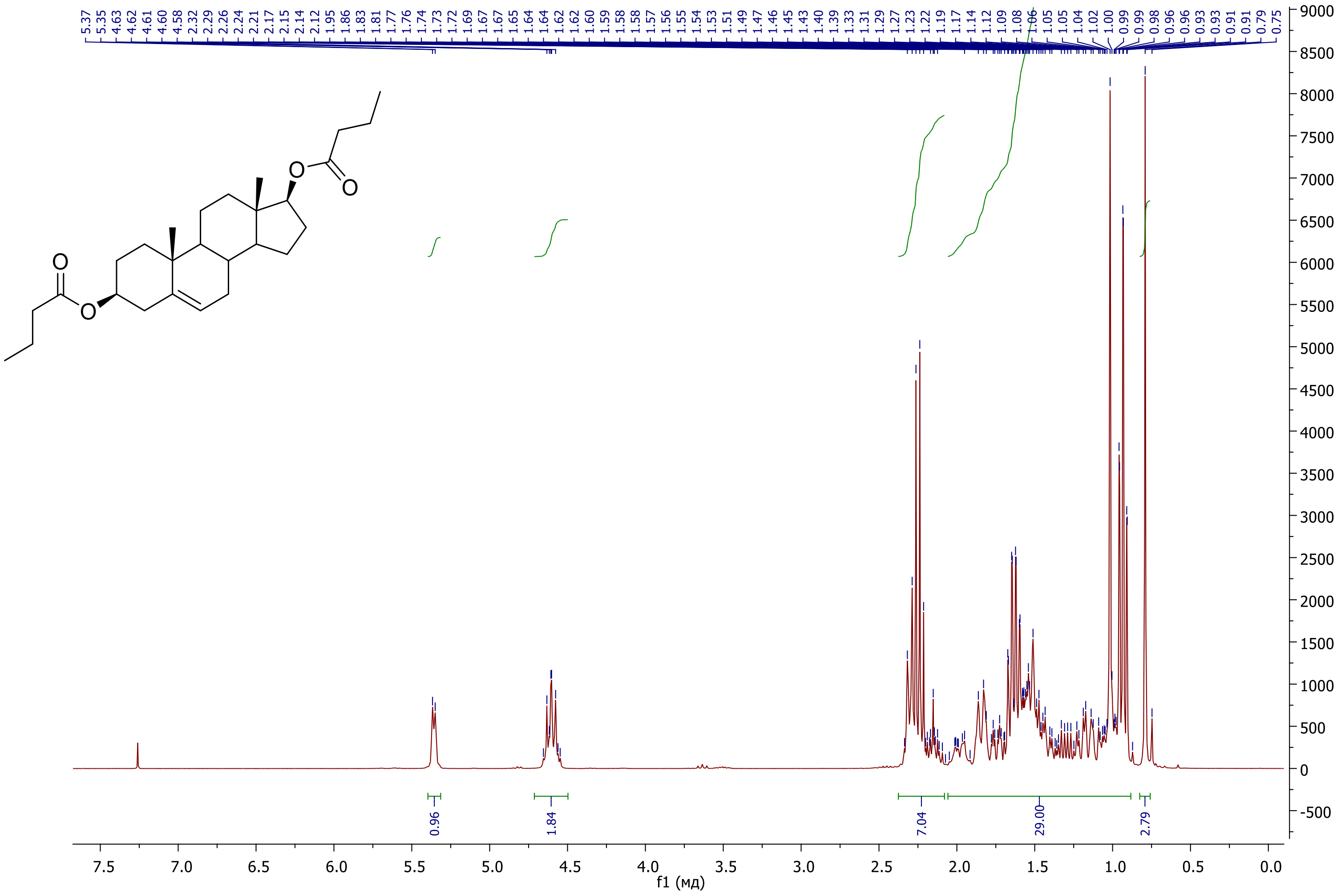

5.37
5.35
4.63
4.62
4.61
4.60
4.58
2.32
2.29
2.26
2.24
2.21
2.17
2.15
2.14
2.12
1.95
1.86
1.83
1.81
1.77
1.76
1.74
1.73
1.72
1.69
1.67
1.67
1.65
1.64
1.64
1.62
1.62
1.60
1.59
1.58
1.58
1.57
1.56
1.55
1.54
1.53
1.51
1.49
1.47
1.46
1.45
1.43
1.40
1.39
1.33
1.31
1.29
1.27
1.23
1.22
1.19
1.17
1.14
1.12
1.09
1.08
1.06
1.05
1.05
1.04
1.02
1.00
0.99
0.99
0.98
0.96
0.96
0.93
0.93
0.91
0.91
0.79
0.75
0.96
1.84
7.04
29.00
2.79
f1 (мд)

4c 13C

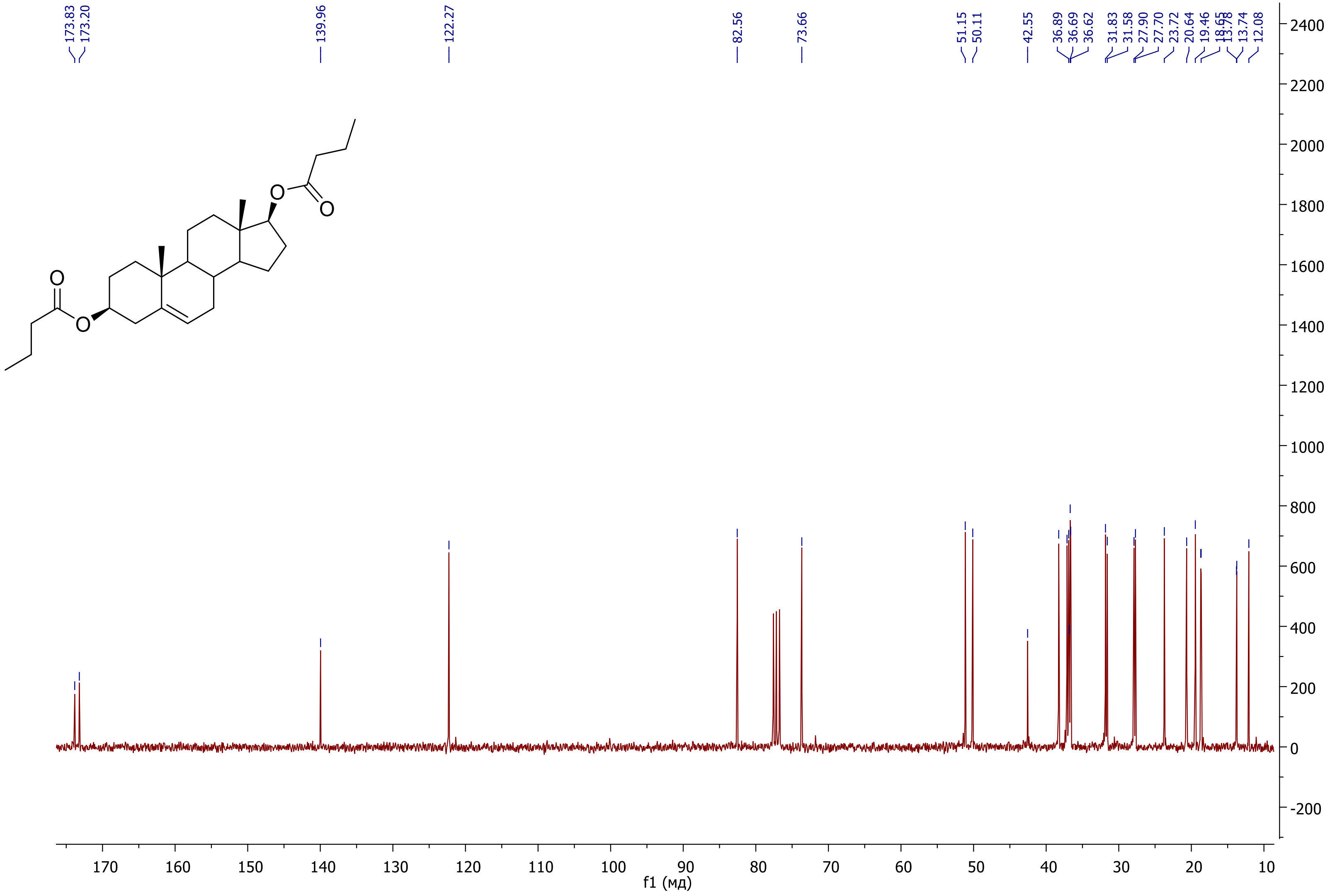
173.83
173.20
139.96
122.27
82.56
73.66
51.15
50.11
42.55
36.89
36.69
36.62
31.83
31.58
27.90
27.70
23.72
20.64
19.46
18.65
13.78
13.74
12.08
f1 (мд)

5 1H

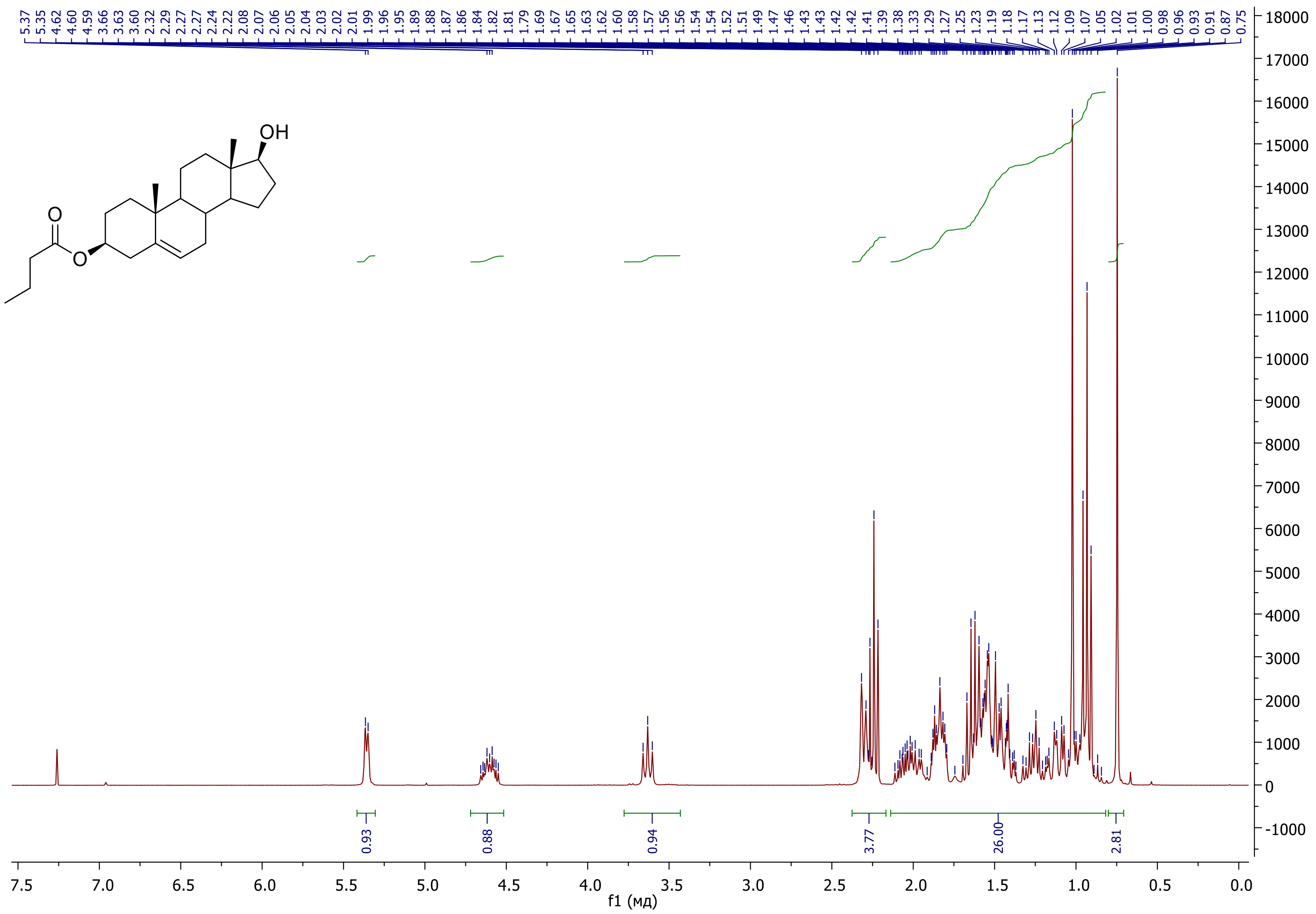

OH
O
O
5.37
5.35
4.62
4.60
4.59
3.66
3.63
3.60
2.32
2.29
2.27
2.27
2.24
2.22
2.08
2.07
2.06
2.05
2.04
2.03
2.02
2.01
1.99
1.96
1.95
1.89
1.88
1.87
1.86
1.84
1.82
1.81
1.79
1.69
1.67
1.65
1.63
1.62
1.60
1.58
1.57
1.56
1.56
1.54
1.54
1.52
1.51
1.49
1.47
1.46
1.43
1.43
1.42
1.42
1.41
1.39
1.38
1.33
1.29
1.27
1.25
1.23
1.19
1.18
1.17
1.13
1.12
1.09
1.07
1.05
1.02
1.01
1.00
0.98
0.96
0.93
0.91
0.87
0.75
0.93
0.88
0.94
3.77
26.00
2.81
f1 (мд)

5 13C

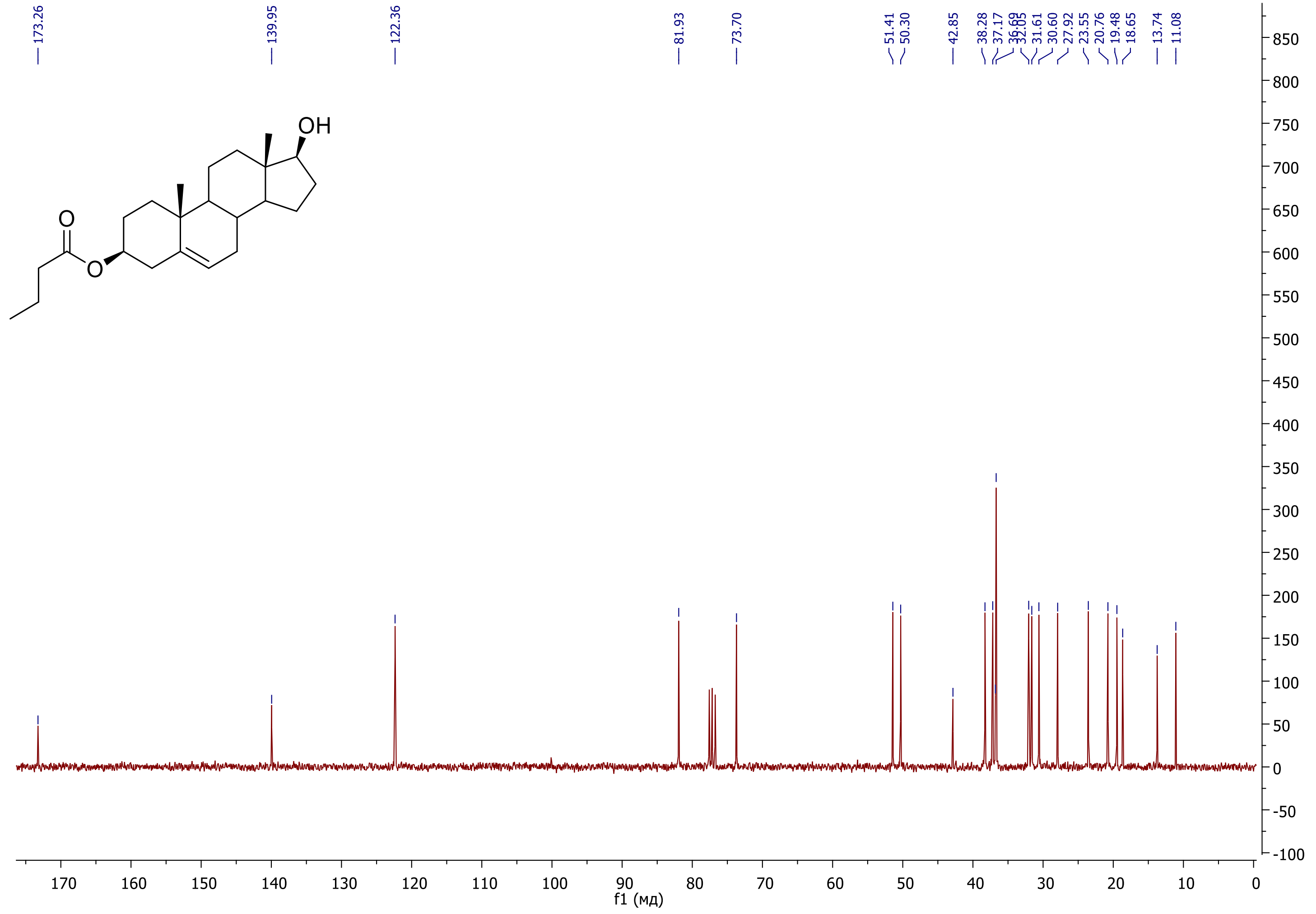

173.26
139.95
122.36
81.93
73.70
51.41
50.30
42.85
38.28
37.17
36.69
32.05
31.61
30.60
27.92
23.55
20.76
19.48
18.65
13.74
11.08
OH
O
O
f1 (мд)
